# Molecular dynamics study on the release of residual stress in milling of 7050 aluminum alloy by ultrasonic treatment

**DOI:** 10.1038/s41598-026-40889-6

**Published:** 2026-02-27

**Authors:** Wenyuan Song, Jinjie Jia, Fei Ma, Yunzhao Zhang

**Affiliations:** Chengdu Aircraft Industrial (Group) Co., Ltd, Chengdu, 610092 China

**Keywords:** Residual stress, Ultrasonic treatment, Molecular dynamics, Dislocation, Engineering, Materials science

## Abstract

Machining deformation caused by the release of residual stress during the processing of large-scale 7050 aluminum alloy thin-walled components remains a critical bottleneck, limiting high-quality and high-precision manufacturing and significantly compromising the dimensional stability of the parts. As a novel stress-control technique, ultrasonic treatment has been proven effective in improving the residual stress distribution within aluminum alloys, thereby reducing the machining deformation of thin-walled structures. However, a systematic analysis of the dynamic evolution and underlying release mechanisms of residual stress under ultrasonic action is still lacking. To analyze defect evolution and the origins of milling-induced residual stress, a molecular dynamics model of milling for 7050 aluminum alloy containing η-phase (MgZn₂ precipitates) was constructed. Following the simulation of the stress release process under ultrasonic treatment, our results reveal that the reduction and homogenization of residual stress are fundamentally attributed to dislocations absorbing energy within the applied ultrasonic field. This energy absorption activates dislocations, enabling them to overcome the potential energy barrier for motion. Consequently, extensive annihilation reactions occur primarily among Shockley partial dislocations, leading to a decrease in the total dislocation density. Ultimately, the internal stress of the material is relaxed and released through plastic deformation mediated by these dislocation reactions.

**Supplementary Information:**

The online version contains supplementary material available at 10.1038/s41598-026-40889-6.

## Introduction

7050 aluminum alloy is widely used in the preparation of complex thin-walled components in the aerospace field due to its low density, high specific strength and good processing performance. In order to meet the requirements of high performance and reliability of aerospace vehicles, 7050 aluminum alloy thin-walled parts are gradually developing in the direction of large size and complexity, which puts forward extremely strict requirements for the size control of products. Thin-walled structural parts usually have a cutting ratio of more than 90%, and have weak stiffness structural features such as multiple cavities and ribs. They are formed after multiple processes such as die forging / rolling, milling / drilling, etc. It is difficult to ensure that they do not deform due to the combined effects of multiple factors^1^.

Among them, the effect of residual stress runs through the whole life cycle of thin-walled blank forming and processing. A large number of studies have proved that the release and redistribution of residual stress are the main deformation reasons of aluminum alloy weak stiffness components^2–4^. When most of the volume of the material is removed, the inherent residual stress in the component will relax and form a new stress distribution state to reach the internal stress balance again, accompanied by a series of deformations such as torsion, warping, and bending. The penetration depth and shape of the stress profile surface of the same batch of blanks will also be different. Even if the same processing parameters are adopted, there will be different distribution of machining residual stress due to different sampling positions^5,6^. Chighizola et al. obtained different initial residual stress fields by using pre-stretching pretreatment and quenching solid solution aging pretreatment on the same aluminum alloy plate respectively. It was found that the amplitude of subsequent processing deformation was positively correlated with the amplitude of initial residual stress, but not with the positive and negative residual stress. The positive and negative residual stress affects the position of the maximum deformation. The different distribution of residual stress in the blank will lead to the change of the amplitude of milling residual stress^7^. The research of Daniel Weber et al. has proved that the maximum contribution of milling residual stress to the deformation of 7050 aluminum alloy frame can reach 82% under different milling schemes. The contribution of residual stress of blank and machining residual stress induced deformation depends on the wall thickness and machining strategy of thin-walled components^8^.Whether the initial residual stress field in the aluminum alloy blank or the residual stress field introduced by the subsequent milling process will inevitably lead to the geometric deformation of the component during the release process, and the machining deformation of the weak stiffness component caused by the residual stress is constantly changing with the process of the process chain. The most difficult problem at present is to improve the deformation by optimizing milling parameters, milling path and clamping force. The lifting space is limited and the residual stress cannot be completely eliminated. In order to suppress the deformation risk caused by residual stress, it is necessary to combine the stress relief treatment process to control the residual stress distribution.

Conventional stress relief process can be divided into mechanical effect method and temperature effect method. The mechanical effect method is to apply static or dynamic stress to the workpiece through mechanical, electromagnetic and other methods to reduce or redistribute the residual stress. It mainly includes vibration aging method, ultrasonic impact method, shot peening method, pre-stretching method, pulse magnetic field method and so on. The temperature effect method is to relax the residual stress until it is eliminated by changing the ambient temperature of the component and adjusting the internal structure under the continuous temperature load, mainly including stress relief annealing, cryogenic treatment and so on. Zhang et al. used ultrasonic vibration aging to eliminate the welding residual stress of aluminum alloy plates. The results show that the maximum residual stress elimination rate of this method can reach 57%. The vibration stress and the initial stress distribution jointly determine the final elimination effect. When the initial residual stress is the same, the larger the vibration ratio, the better the stress elimination effect^9^. Yan et al. carried out the elimination test of rolled magnesium alloy by pulsed magnetic field method. Under the action of Lorentz force, dislocations began to move to induce local plastic deformation, and the maximum residual stress elimination rate after treatment was 30.3%^10^. Tang et al. studied the elimination effect of residual stress and the change of mechanical properties of AlSi10Mg alloy under different aging temperatures. It was found that the crystal structure, size and Si atom expansion mode of the material changed under direct aging, which led to the change of yield strength, tensile strength and elongation. The maximum release rate of residual stress after treatment was 32%^11^. Wen et al. conducted a stress relief test on the 7050 Al alloy bar after laser shock processing by cryogenic treatment combined with low temperature tempering. It was proved that the combined process of cryogenic treatment and supplementary low temperature tempering can reduce the compressive stress on the surface introduced by laser shock and reduce the hardness difference between the matrix and the impact surface^12^.

Although various experimental studies have proved the effect of conventional stress relief processes, these techniques also have certain limitations when applied to the residual stress control of thin-walled aluminum alloy components. As the conventional vibration aging method is carried out under the low-order vibration mode of the component, it is difficult to introduce uniform vibration stress for large-scale complex thin-walled components with lower first-order vibration frequency, and the local position may be deformed after treatment. The area treated by strengthening processes such as ultrasonic impact and shot peening is the surface of the material. If not handled properly, it may also introduce micro-cracks on the surface of the material due to long-term alternating load, which will cause damage to the product quality. Similarly, the use of aging method and stress relief annealing to eliminate residual stress at the same time, there will be a high temperature to reduce the strength of the material, low temperature to remove residual stress effect is poor. Although cryogenic treatment can effectively reduce some residual stress peaks, it cannot accurately control the overall residual stress distribution. For thin-walled components, there are problems such as larger deformation after treatment, more complex deformation of the profile and difficult to control.

With the accumulation and iteration of various stress relief methods and techniques, a series of new residual stress control methods have been gradually derived. The research shows that the reduction and homogenization of residual stress in this area can be realized by ultrasonic control of the original blank or the thin-walled parts before and after machining^13^. Many scholars have explored the application scenarios of ultrasonic regulation of residual stress in the actual processing, manufacturing and service process. Guo et al. compared the effect of eliminating residual stress on the machining of aluminum alloy box cover. The results show that the ultrasonic regulation method has a local stress elimination rate of 88.7%, which is better than the spectrum harmonic method, and the diameter deformation of the part after regulation is less than 10μm^14^. Jin et al. realized the machining deformation control of titanium alloy spinning tube components through ultrasonic control technology, and finally realized the control of the roundness of the easy-to-deform nozzle of the finished component by reducing and homogenizing the residual stress of the blank and the residual stress of rough machining and semi-finishing^15^. In order to solve the potential safety hazards such as deformation and instability of components caused by the release of welding residual stress during the service of nuclear power steel containment, Xiao et al. carried out ultrasonic treatment on the weld area. After treatment, the residual tensile stress decreased by 21 MPa on average, and the stress control effect was equivalent to that of traditional heat treatment after holding for 10 hours^16^. At present, the research on the effect of ultrasonic treatment on residual stress distribution mainly focuses on the effectiveness verification of control method on the reduction and homogenization of residual stress such as casting, welding and additive manufacturing, and how to optimize the design control process scheme and develop ultrasonic control system suitable for complex thin-walled structure characteristics. The mechanism of residual stress control is not clear, and there is a lack of research and analysis on the continuous process from the introduction of residual stress in milling to the release of residual stress by ultrasonic treatment.

Directly revealing the mechanism of residual stress release through experimental methods alone is often prohibitively expensive and challenging. In contrast, computational simulation offers a powerful alternative by providing atomic-scale evolution information that is difficult, if not impossible, to obtain from macroscopic milling experiments. This approach allows for the analysis and understanding of both the milling process and resultant residual stress from a microscopic perspective. The theoretical insights gained can then be refined through experimental verification, thereby facilitating the development of ultrasonic treatment technology. Therefore, this study establishes a molecular dynamics model of the milling process for 7050 aluminum alloy. First, the model simulates residual stress evolution following diamond blade milling of a planar component, analyzing the influence of dynamically changing micro-defects on stress development and elucidating the formation mechanism of post-milling residual stress. Subsequently, it simulates the release process of this milling-induced residual stress under ultrasonic regulation, discussing the concomitant evolution of internal stress and dislocation defects. This work explains the underlying physical mechanism of the ultrasonic regulation process. Furthermore, the predicted macroscopic trend of residual stress reduction is validated experimentally. Collectively, this study provides theoretical support for controlling milling-induced residual stress and deformation in 7050 aluminum alloy aviation thin-walled parts.

## Models and methods

To guide the optimization of processing parameters and provide theoretical support for high-precision and high-efficiency machining, it is essential to analyze the mechanisms through which residual stress is introduced during milling and subsequently released under ultrasonic treatment. To fully capture the dynamic changes of milling-induced residual stress under ultrasonic action, a continuous simulation approach is employed using the LAMMPS software. Initially, a molecular dynamics model of 7050 aluminum alloy containing an MgZn₂ precipitate is established to simulate the process of groove formation via single-point diamond cutting. Subsequently, a periodic vibration is applied to this model containing the as-milled residual stress field to simulate the macroscopic ultrasonic treatment process, thereby allowing for analysis of the residual stress release mechanism.

### Initial modeling of 7050 Al alloy

The main elements of 7050 aluminum alloy are Zn and Mg. When magnesium is added to the zinc element of 3–7.5%, the MgZn_2_ phase will be formed. Martin Vlach et al^17^. proved that the aging precipitation sequence of 7-series aluminum alloy is: Mg clusters or coherent region (GP zone) transformed into $\eta{\prime}$ metastable phase (MgZn2, semi-coherent ), and finally transformed into stable phase $\eta$ (MgZn2, incoherent). Yu et al.also proved that the phase is the main strengthening phase in 7-series aluminum alloy by experimental observation^18^. Previously, the molecular dynamics study of aluminum alloys mostly used the face-centered cubic (FCC) crystal structure of pure aluminum matrix for cutting analysis. It is necessary to establish a model containing the η-phase in order to analyze the micro-defects/microstructure evolution in the milling process more comprehensively. The references^19,20^ have shown that in the molecular dynamics calculation, the precipitated phase is placed in the matrix atom in the form of ' mosaic ', which can reliably simulate the elastoplastic behavior and thermodynamic properties of multi-component materials. Therefore, in this paper, 7050 aluminum alloy is studied with aluminum atom as the matrix, and stable η-phase is added inside it to establish a milling model. All the microscopic models and dislocation reaction pictures are exported by OVITO software (Open visualisation tool) and marked in VISIO.

**Fig. 1:**
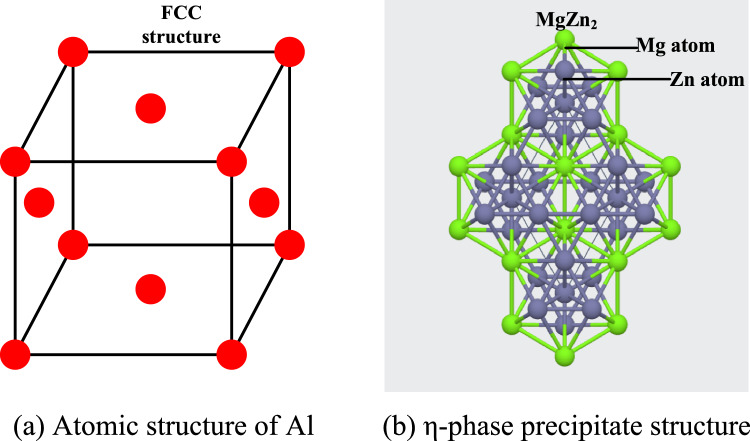
Cell structure of Al atom and η-phase precipitate. (**a**) Atomic structure of Al. (**b**) η-phase precipitate structure.

**Fig. 2:**
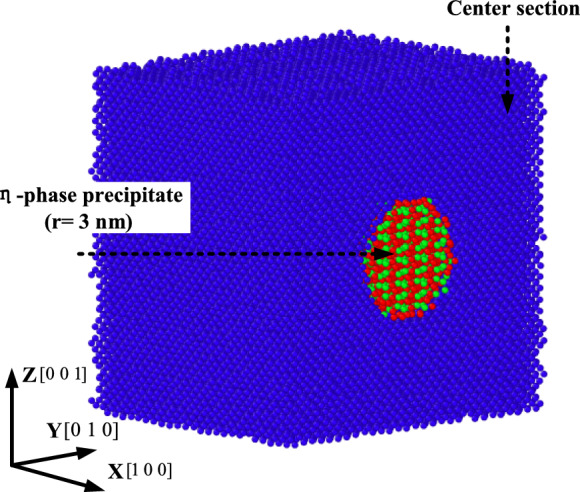
Construction of initial aluminum alloy model.

As shown in Fig. 1a, the single crystal aluminum matrix is FCC crystal structure, there is an atom at the vertex and face center of its unit cell, and the η-phase precipitate belongs to the hexagonal crystal system, as shown in Fig. 1b. The space group is P6_3_/mmc((No. 194)), Mg atoms occupy the 2a site (0,0,0;0,0,1/2), and Zn atoms occupy the 4f. site (1/3, 2/3, z; 2/3, 1/3, z + 1/2; …z = 0.056). The parameters of various crystal structures are shown in Table 1. Using the open source modeling software Atomsk, the rectangular aluminum matrix is first constructed, and then the spherical MgZn_2_ particles are merged into it to realize the modeling of the initial 7050 aluminum alloy, as shown in Fig. 2.

**Table 1:** Lattice parameter.

Cell type	Lattice constant a	Lattice constant b	Lattice constant c	Angle of edges α	Angle of edges β	Angle of edges γ
Al (FCC)	4.050 Å	4.050 Å	4.050 Å	90°	90°	90°
MgZn_2_	5.251 Å	5.251 Å	8.445 Å	90°	90°	120°

### Milling simulation settings

**Fig. 3:**
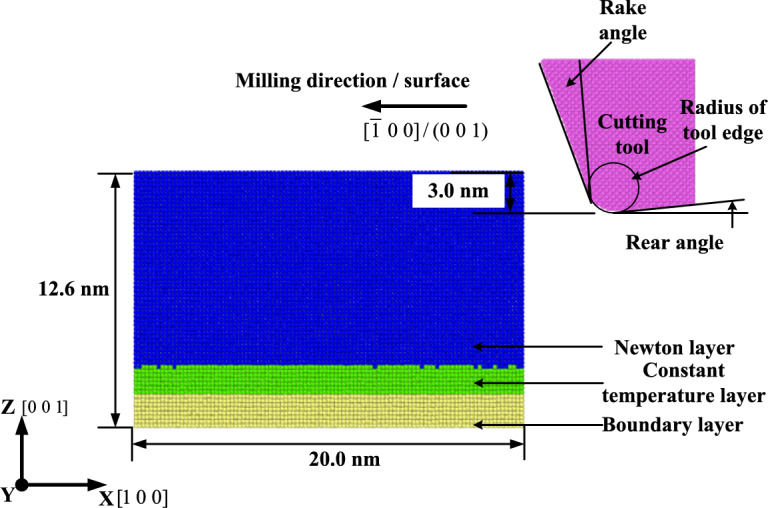
Milling model.

The cutting model is shown in Fig. 3. The aluminum alloy atoms and diamond atoms in the model are about 180,000 and 55,000 atoms. The workpiece model is divided into Newton layer, constant temperature layer and boundary layer. Among them, the boundary layer atom with a thickness of 10 Å at the bottom is set to a fixed state to prevent the material from moving during milling, while the thermostatic layer between the Newtonian layer and the boundary layer simulates the heat exchange between the material and the outside world. The temperature is always maintained at 300 K by the velocity calibration method. When the temperature exceeds 300 K, the atomic velocity through the scaling velocity thermostatic layer keeps the temperature constant. In order to avoid the influence of size effect as much as possible, the Y $[0{ 1 0}]$ direction is set as a periodic boundary condition. The grinding of the tool is not considered, so in order to simplify the calculation, the whole tool is set as a rigid body in the simulation. At the beginning of milling, the model is relaxed to minimize the initial energy. The simulation parameters are shown in Table 2.

**Table 2:** Milling simulation parameters.

Simulation parameters	Setting parameter
Workpiece size	20 nm $\times$ 12 nm $\times$ 12.6 nm
Radius of tool edge	1 nm
Rake angle	20°
Rear angle	5°
Milling speed	0.08 nm/ps(80 m/s)
Time step	1 fs

The potential function settings involving the elements are shown in Table 3. The MEAM potential function file is derived from the open source potential function website NIST ( www.ctcms.nist.gov/potentials/), and the L–J potential function is obtained from the Ref 28. These potential functions have been shown to correctly simulate the defect evolution and deformation of the related atoms at the microscopic scale^21–25^. The continuum hypothesis in macroscopic mechanics has failed in molecular dynamics calculations. At the microscopic scale, Viral stress is usually used to calculate the average stress of the atomic system $\prod^{\alpha \beta }$^26^:

1 $$\prod^{\alpha \beta } = \frac{1}{\Omega }\left[ { - \sum\limits_{i} {m_{i} v_{i}^{\alpha } v_{i}^{\beta } } + \frac{1}{2}\sum\limits_{i} {\sum\limits_{j \ne i} {F_{ij}^{\alpha } r_{ij}^{\beta } } } } \right]$$

**Table 3:** Atomic potential function settings.

Atomic form	Potential function	global cutoff for LJ	global cutoff for Coulombic
Al–Mg–Zn	MEAM^28^	–	–
C–Mg	Lennard–Jones	0.0046	3.21
C–Al	Lennard–Jones	0.048	3.305
C–Zn	Lennard–Jones	0.0048	3.31

In the formula 1, $\Omega$ is the total volume of the ensemble, $\alpha ,\beta$ is the Cartesian coordinate, $F_{ij}$ is the force between the *i* th atom and the *j* th atom, and $r_{ij}$ is the distance between the two atoms. The first term on the right side of the equal sign is derived from the thermal vibration in the system, which is equal to zero in the static simulation. The second term is derived from the interaction between atoms and is considered to be the average of Cauchy stress.

The stress tensor of any atom i in the model can be expressed as^27^:

2 $$\sigma_{ij} = \left( {\begin{array}{*{20}c} {a_{11} } & {a_{12} } & {a_{13} } \\ {a_{21} } & {a_{22} } & {a_{23} } \\ {a_{31} } & {a_{32} } & {a_{33} } \\ \end{array} } \right)$$

Among them, $\sigma_{11}$, $\sigma_{22}$ and $\sigma_{33}$ are normal stresses, while the remaining components are shear stresses.

Affected by the size effect of molecular dynamics, the stress in the simulation process is actually microscopic residual stress. In order to compare with the average volume residual stress detected by ultrasonic measurement method in the macroscopic test in the 5 Section, the residual stress in the microscopic simulation is defined as the hydrostatic stress after the model relaxation^29^. The model is layered with equal thickness along the Z direction, and the average residual stress $\sigma_{micrs}$ in each layer is determined by the average normal stress in the three directions:

3 $$\sigma_{micrs} = {\raise0.7ex\hbox{$1$} \!\mathord{\left/ {\vphantom {1 3}}\right.\kern-0pt} \!\lower0.7ex\hbox{$3$}} \cdot [\sigma_{11} + \sigma_{22} + \sigma_{33} ]$$

Since the output stress in LAMMPS software is the volume multiplied by the pressure, the output file needs to be post-processed after simulation. The normal stress of all atoms in each residual stress calculation area is counted and output, and the total atomic volume in the area is divided to obtain the microscopic residual stress value. The stress change during the machining process is analyzed by Von Mises, and the calculation formula is as follows:

4 $$\sigma_{von} = \sqrt {\frac{1}{2}[(\sigma_{11} - \sigma_{22} )^{2} + (\sigma_{11} - \sigma_{33} )^{2} + (\sigma_{33} - \sigma_{22} )^{2} ] + 3(\sigma_{12}^{2} + \sigma_{13}^{2} + \sigma_{23}^{2} )}$$

### Ultrasonic control parameter setting

Studies have shown that the softening effect of macroscopic high-energy ultrasound is proportional to the acoustic energy density^30^, and the relationship between the decrease of flow stress and the acoustic energy density can be expressed as^31^:

5 $$\Delta \tau_{{\mathrm{f}}} = {\mathrm{c}}_{1} E_{acoustic}^{{{\mathrm{c2}}}}$$

Among them, c1 and c2 are the material parameters obtained from the test, $E_{acoustic} = {\mathrm{A}}_{u}^{2} \omega^{2} \rho$ is the sound energy density, and is related to the square of the vibration amplitude ${\mathrm{A}}_{u}^{{}}$, the square of the angular frequency $\omega$ and the material density. With further experimental research and analysis, Cheng et al.^32^ found that the ultrasonic softening effect of 2 series and 7 series aluminum alloys is inversely proportional to the volume, while the actual ultrasonic softening effect is related to the amplitude. The control process of applying an excitation to the ultrasonic transducer in close contact with the metal component on the macroscopic scale can be regarded as the vibration behavior of the atom subjected to periodic external loads. Zhao et al.^33^ reproduced the ultrasonic softening phenomenon of single crystal / polycrystalline copper atoms during tension by applying a fixed amplitude periodic atomic vibration simulation in the loading direction. The calculation results are in good agreement with the macroscopic tensile results, and the dislocation nucleation, proliferation, movement and annihilation evolution during plastic deformation are fully demonstrated.

In this paper, atomic vibration is used to simulate the ultrasonic regulation process of 7050 aluminum alloy which is excited by transducer and releases stress. Due to the size effect of molecular dynamics, in order to improve the computational efficiency, it is necessary to use the similarity principle to scale the vibration parameters^34^. The minimum wall thickness size of aluminum alloy weak stiffness structural parts is usually in the order of $1 \times 10^{ - 3}$ m, while the size of the model in this study is $1 \times 10^{ - 8}$ m, which is about 10^5^ orders of magnitude different from the size of the actual thin-walled structure. The no-load amplitude of the single-channel transducer used in practical engineering applications is $1 \times 10^{ - 5}$ m. The macroscopic amplitude is reduced in equal proportion, and the amplitude applied on each atom is 3 Å. The longitudinal wave velocity cl excited by the transducer with a center frequency of f satisfies ${\mathrm{c}}_{l} = \lambda_{l} f$ in the material, so the wavelength $\lambda_{l}$ is also scaled based on the similarity principle. When the actual transducer frequency is ≥ 20 kHz, the corresponding analog frequency should be ≥ $2 \times 10^{6}$ KHz. The ultrasonic vibration period was set to 0.5 ps, and a total of 80 ps was regulated. In the simulation, the atomic vibration is realized by the fix move wiggle command. Before the initial simulation, the boundary conditions of the model are set to periodic boundary conditions to reduce the influence of size effect. Before the regulation, the whole model is relaxed to achieve internal stress balance. After the regulation, the relaxation 5 ps treatment is carried out to rebalance the internal stress and form the residual stress distribution.

## Analysis of the generation process of milling residual stress

### Atomic motion law in milling process

**Fig. 4:**
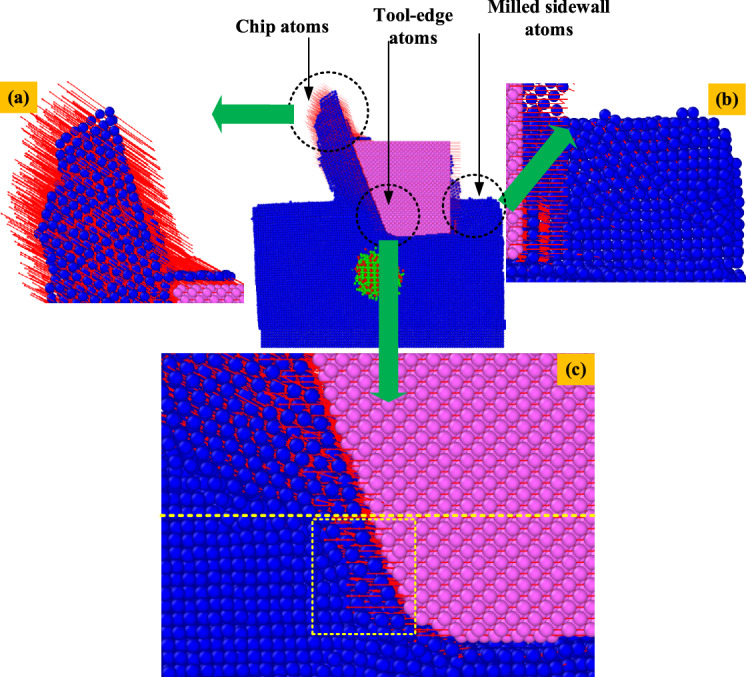
Atomic displacement vector: (**a**) Chip atoms; (**b**) Milled sidewall atoms; (**c**) Cutting tool front atom.

Taking the stable motion state when the tool completely moves into the center of the material as an example, the atomic motion trajectory of the center XOZ section in the width direction is plotted as a vector diagram as shown in Fig. 4. In the milling process, the effect of the tool on the surface atoms of the material has both extrusion and shear effects. In various macroscopic experimental analysis, it has been proved that the tool with a relative sharpness (tool edge radius / milling depth) less than 1 acts on the material when the shear effect is dominant. The relative sharpness of the tool model established by simulation is 1/3, which is consistent with the macroscopic cutting phenomenon. The effect of the tool on the matrix atom is mainly reflected in the shear force.

The essence of nano-scale milling is the process of material atom migration and discrete motion under the action of tool. When the shear effect is dominant, there is a high material removal rate. The atoms on the surface of the workpiece undergo severe plastic deformation due to shear and extrusion. With the action of high temperature and high pressure, they adhere to the tool to form chips, which are discharged from the matrix along the movement direction of the workpiece (Fig. 4a). Most of the atoms in the side wall surface of the finished milling are also sheared by the tool and tend to move upward in the tangential direction, which makes the upper surface layer produce an irregular arrangement. A small number of atoms at the bottom of the side wall move forward. The red arrow is the motion vector of the atom. A small number of atoms in the box below the boundary line are squeezed forward by the tool, while most of the atoms above the yellow line are inclined upward by the shear force provided by the tool. The atoms crossed by the yellow dotted line hardly move, forming a stagnation point^35^.

### Analysis of plastic deformation behavior in milling

**Fig. 5:**
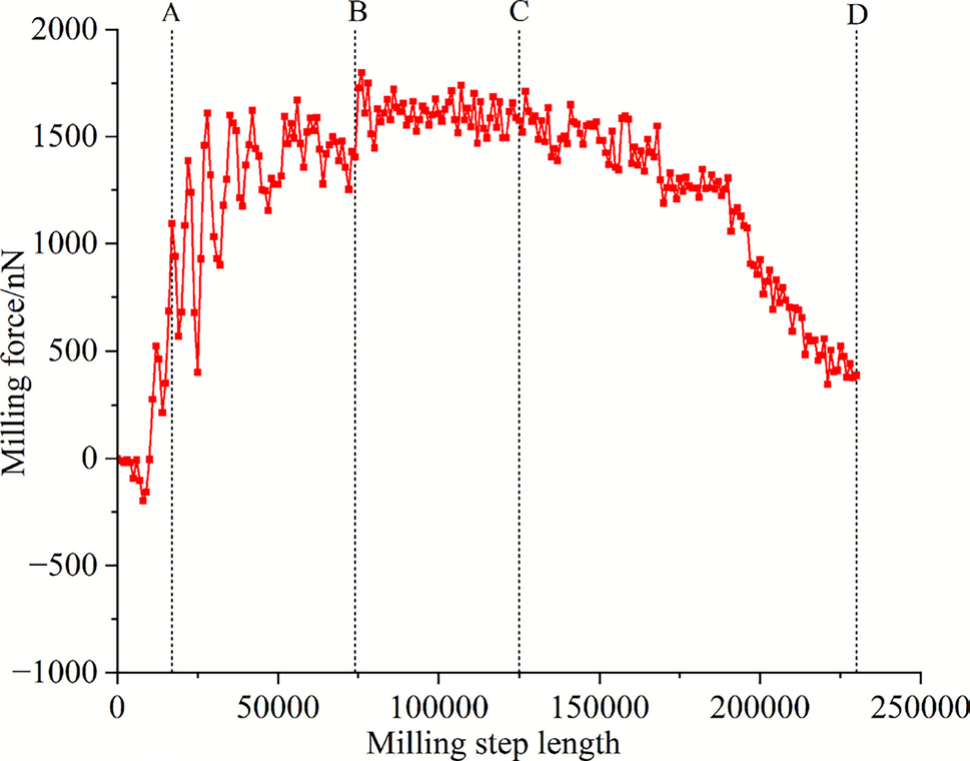
Milling force variation in the milling direction.

In order to intuitively reflect the relationship between the plastic deformation behavior in the milling process and the tool motion, the milling force change of the tool along the milling path during milling is first drawn in Fig. 5. When the initial tool is not in contact with the workpiece, the milling force is 0, and when the initial contact material, the milling force curve produces a very short ' hook ' shape interval, that is, a negative milling force appears, which is generated by the attraction between the diamond atom and the matrix aluminum atom. After entering the normal milling state, the milling force rises sharply, and then enters a relatively stable state, until the milling force gradually begins to decline in the later stage of milling.

**Fig. 6:**
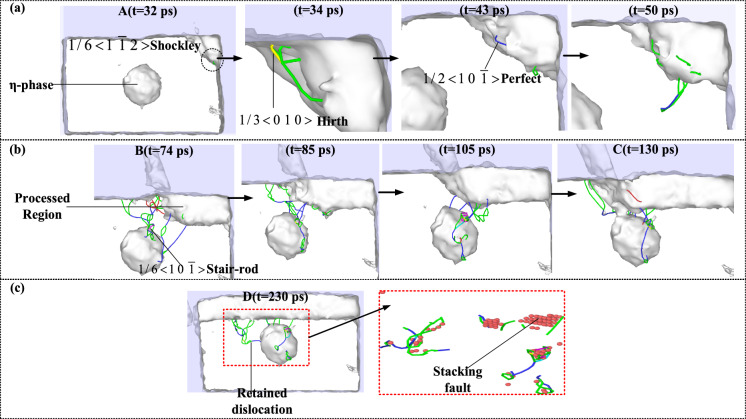
Plastic deformation behavior in milling process.

To further analyze the plastic deformation behavior in the milling process, four typical stages of milling force change were selected to visualize the dislocation and defect atoms by DXA analysis function, as shown in Fig. 6. When the tool moves to point A, the lattice distortion at the front end of the tool reaches a critical value, and the Shockley partial dislocation with a smaller Bernoulli vector first nucleates and moves along the $(1{ 1 1)}$ plane. Subsequently, a large number of Shockley partial dislocations and a small number of Hirth dislocations and Perfect full dislocations were generated, which made the material exhibit work hardening phenomenon, so the milling force of the tool continued to rise before the stable milling stage. After the two Shockley partial dislocations meet during this period, the annihilation reaction of Eq. 6 occurs because the Bohr vector b is equal and the direction is opposite, which constitutes a certain dynamic recovery process:

6 $$\frac{1}{6} < 1\overline{1}2 > + \frac{1}{6} < \overline{1}1\overline{2} > = 0$$

As shown in Fig. 6 (b), when entering the stable milling state, the first dislocation moves continuously to the $(\overline{1} \overline{1} 1)$ plane until it meets with η-phase, and the dislocation reaction of Eq. 7 produces the Stair-rod dislocation:

7 $$\frac{1}{6} < 2\overline{1}1 > + \frac{1}{6} < \overline{1}1\overline{2} > = \frac{1}{6} < 10\overline{1} >$$

Since the Stair-rod dislocation is an immovable dislocation, it will form a pinning effect on the surrounding dislocations to hinder their movement. The Hirth dislocation does not only climb on the slip surface, and the strengthening effect of the η-phase precipitates will hinder the dislocation movement and improve the strength of the material. Therefore, the continuously generated dislocations accumulate between the tool and the η-phase. Moreover, the helical winding connection of the retained multiple dislocations constitutes the Lomer-cottrell dislocation lock, which further inhibits the movement of other dislocations. At the same time, with the increase of milling distance, huge energy is provided under the coupling effect of milling force and milling heat to make the interior cross the energy barrier and form more defect structures. As shown in Fig. 6c, the dislocations trapped below the milling layer are stored in the lattice in the form of strain energy at the end of milling. When the strain energy accumulates to a certain extent, it is released, which promotes the transformation of the crystal structure. The main behavior of plastic deformation in milling is that a large number of dislocations dominated by Shockley partial dislocations are retained inside the workpiece, and stacking faults are formed around the dislocations.

**Fig. 7:**
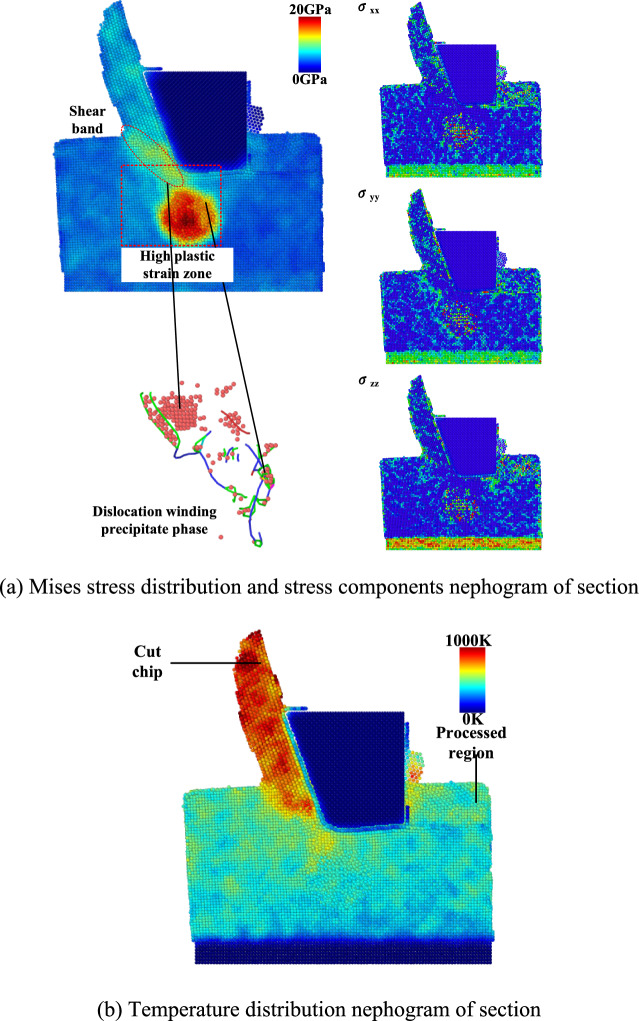
Von Mises stress and temperature distribution cloud diagram when milling to the center position. (**a**) Mises stress distribution and stress components nephogram of section. (**b**) Temperature distribution nephogram of section.

The Von Mises stress cloud diagram and temperature distribution of the XOZ section when milling to the center are drawn as Fig. 7. When milling to the center of the length, a shear band with higher Mises stress is gathered at the front end of the tool edge. Under the action of large strain energy, the local lattice is directly destroyed, and a high plastic strain zone is formed below it. At this moment, the atoms in the high plastic strain zone enter the athermal hardening stage, the work hardening rate remains almost unchanged, and the slip system of multiple dislocations starts with a large number of dislocation multiplication, which is strengthened and hindered by the central η-phase. The effect is wrapped around it and a large number of stacking fault atoms are formed below the front end of the tool.

The dislocation needs to absorb enough energy to slip and climb across the barrier. The total energy $\Delta {\mathrm{F}}^{dis}$ required to overcome is composed of mechanical work $\Delta {\mathrm{W}}^{dis}$ and Gibbs free energy $\Delta G^{dis}$, as shown in Eq. 8:

8 $$\Delta {\mathrm{F}}^{dis} = \Delta {\mathrm{W}}^{dis} + \Delta {\mathrm{G}}^{dis}$$

The higher temperature in the high plastic strain region gives the atoms enough Gibbs free energy, which further increases the aggregation reaction of dislocations. The atoms above 500 °C are concentrated in the chip area in front of the tool. The temperature of the milling surface area and the internal atoms is lower than that of the chip. This is due to the fact that all the atoms in the Newton layer in the NVE simulation ensemble cannot directly dissipate heat, and need to rely on the constant temperature layer at the bottom of the material for heat exchange. The farther away from the constant temperature layer, the more limited the heat transfer of the atoms. The atoms generated under the action of high temperature move along the chip to the top of the tool, which reduces the thermal damage of the machined area to a certain extent.

### Analysis of milling residual stress distribution law

**Fig. 8:**
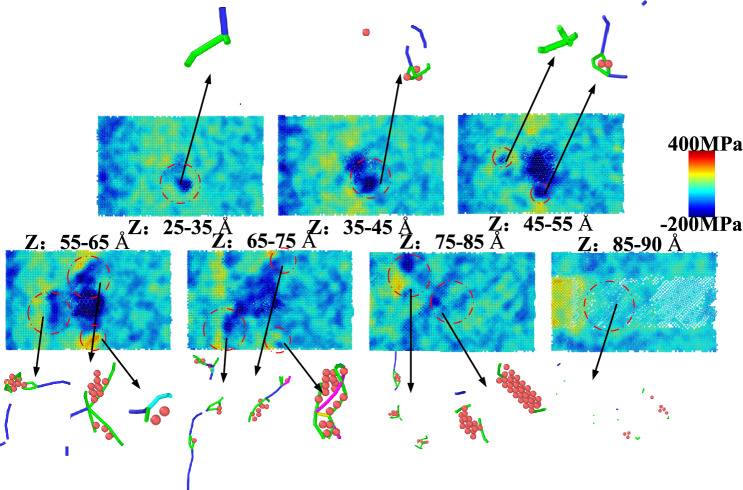
Newton layer residual stress distribution diagram.

**Fig. 9:**
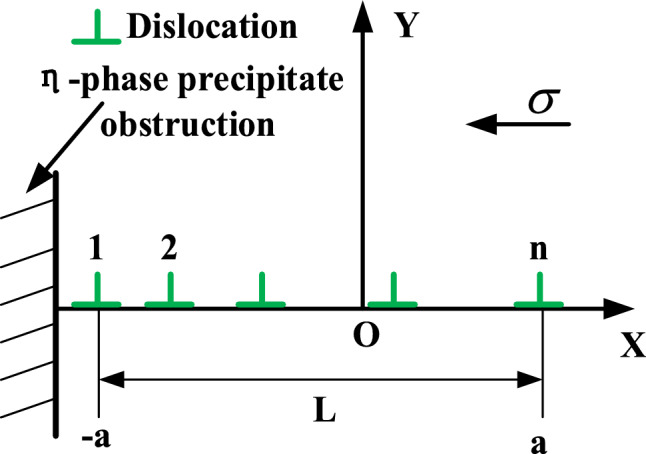
Schematic diagram of dislocation pile-up.

The atoms of the Newton layer are layered when the milling is completed, and the microscopic residual stress (hydrostatic stress) contained in it is dyed and the corresponding defect distribution in each layer is drawn as shown in Fig. 8. Since there is a spherical η-phase with a diameter of 40 Å at the center of the initial model, the coherent precipitated phase is subjected to compressive stress due to the attraction of the matrix atoms. Therefore, it can be clearly observed that a circular compressive stress concentration area is generated at the center of the cross section within the thickness range of 35–75 Å. After milling, the stress balance of the upper atoms is broken, so that there is a relatively uniform stress distribution in the milling surface layer (Z = 85–90 Å). The original precipitation of the corresponding stress concentration area in the near surface layer (Z = 75–85 Å) is broken. The balance is re-evolved into a cross-sectional distribution of tensile stress in the mixed part dominated by compressive stress. The position of dislocation retention in each section is the stress concentration area, which also shows that the local area has severe plastic deformation and the residual stress stays in the Newton layer in the form of elastic energy. Dislocations generated by milling external loads cannot pass through and are arranged in sequence when encountering η-phase obstacles. Dislocations released by the same dislocation source will also be hindered by other dislocations. This field is called dislocation pile-up, as shown in Fig. 9^36^. The number of pile-up dislocations in front of the η-phase is n, and the two-dimensional coordinate center is defined to be located at the center of the pile-up group. The total length of these dislocations is the length L of the pile-up group. When the first No.1 dislocation is hindered and stops moving, it will be subjected to the force of other dislocations and external loads. The total force $F_{{\text{dis - j}}}$ of the* j*th dislocation in the whole dislocation pile group can be expressed as:

9 $$F_{{{\mathrm{dis}} - {\mathrm{j}}}} = \frac{{Gb^{2} }}{2\pi K}\sum\limits_{\begin{subarray}{l} i = 1 \\ j \ne i \end{subarray} }^{n} {\frac{1}{{x_{j} - x_{i} }}} + \sigma b$$

There are n dislocations in the plug group. According to the principle of virtual work, the force of the plug group dislocation on the obstruction can be calculated: Suppose that under the action of external load, the No.1 dislocation will hinder the distance of the object to push forward $\delta x$, then the remaining dislocations will also move forward the same $\delta x$, and the work done by the external force on the plug group dislocations is $\sigma nb\delta x$. At this time, let the force between the No.1 dislocation and the hindrance be $\sigma_{dis - 1}$, then the work done by the hindrance to it is $\sigma_{dis - 1} b\delta x$. The external load and the hindrance have the same force on the whole plug group, that is:

10 $$\sigma_{dis - 1} b\delta x = \sigma nb\delta x$$

Therefore, $\sigma_{dis - 1} = \sigma n$ is known, indicating that the external force on the dislocation pile-up is related to the number of dislocations in the pile-up group. Usually, when the dislocation emitted by the dislocation source is hindered by both sides, the bilateral pile-up effect on both sides of the dislocation will be triggered, that is, dislocations of length a are accumulated at both ends of the coordinate center. For a continuous plug group, the dislocation density can be expressed as:

11 $$n(x) = \pm \frac{1}{b}\frac{db(x)}{{dx}}$$

In the formula, $\pm$ represents the direction of the dislocation. When the dislocation is balanced, the sum of the forces is transformed into an integral form as follows^36^:

12 $$\sigma b - \int\limits_{ - a}^{ + a} {\frac{{\mu b^{2} }}{2\pi (1 - v)}} \frac{{n(x{\prime} )dx{\prime} }}{{x{\prime} - x}} = 0$$

The total number of dislocations N is :

13 $$N = \int\limits_{0}^{a} {n(x)dx} = \frac{2\sigma (1 - v)}{{\mu b}}\int\limits_{0}^{a} {\frac{xdx}{{(a^{2} - x^{2} )^{1/2} }}} = \frac{\sigma (1 - v)L}{{\mu b}}$$

Equation 13 indicates that a greater length L of the dislocation pile-up corresponds to a larger number N of dislocations within the pile-up group. Consequently, a higher density of dislocations blocked within the matrix after milling leads to more pronounced stress concentration. This theoretical relationship is consistent with the residual stress distribution observed in Fig. 8. In summary, the milling-induced residual stress originates from the evolution of dislocations under the coupled effects of mechanical and thermal loads, with the mechanical load playing the dominant role. At the microscale, the primary manifestation of this residual stress is the plastic deformation caused by dislocation pile-ups. This is further influenced by the interaction between these pile-ups and the η-phase precipitates within the material, which collectively correspond to the regions exhibiting significant stress concentration in the simulation.

## Analysis of milling residual stress release process under ultrasonic treatment

**Fig. 10:**
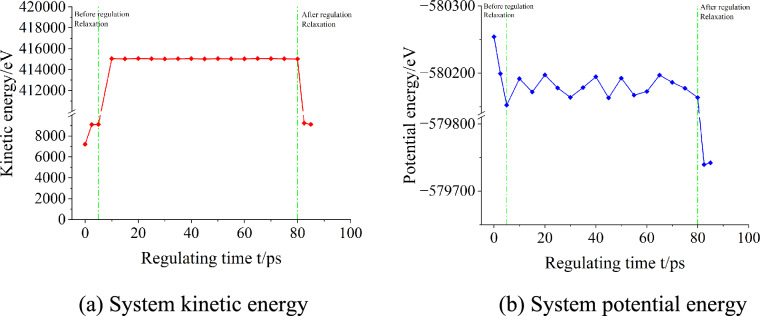
The change of kinetic energy and potential energy in the whole process of ultrasonic regulation. (**a**) System kinetic energy. (**b**) System potential energy.

From the analysis of the Sect. 3, it can be seen that the position of dislocation aggregation at the end of milling forms a severe plastic deformation zone, and the residual stress is retained in the material in the form of elastic energy. Therefore, the line defects dominated by uneven dislocation distribution in the whole component lead to the generation of milling residual stress. The changes of kinetic energy and potential energy inside the material during the regulation process are shown in Fig. 10. The kinetic energy of the material increased slightly by relaxation treatment before regulation, and reached the balance of kinetic energy at 2.5 ps. Since the potential function is used to describe the interaction between atoms in molecular dynamics simulation, it can be seen from the potential function that when the atomic distance is less than the truncated radius ${\mathrm{r}}_{0}$, the force is repulsive, and when it exceeds the truncated radius range, it is gravitational. It is considered that when the two atoms are infinitely far away from each other, it is the potential energy 0 point. Therefore, the ' negative sign ' of the calculated potential energy indicates that the interaction between the atom and the relative infinity is approximately zero gravity. The relaxation treatment before regulation reduces the potential energy of the material accordingly, so that the total energy of the system reaches equilibrium. After applying high-energy ultrasound to the milled aluminum alloy material, the internal atomic kinetic energy increases sharply, and then tends to be stable during the control process. The retained dislocations in the components after milling are stored in the form of elastic potential energy field, so the potential energy of the whole system will fluctuate with the reaction of dislocations during the regulation process. After the end of the regulation, with the sudden stop of energy input, the kinetic energy in the whole system also decreases sharply again. Due to the decrease of dislocation density after the regulation, the potential energy of the system also decreases, so that the total energy decreases and is in a stable state of low energy.

### Dynamic evolution of internal stress under ultrasonic regulation

**Fig. 11:**
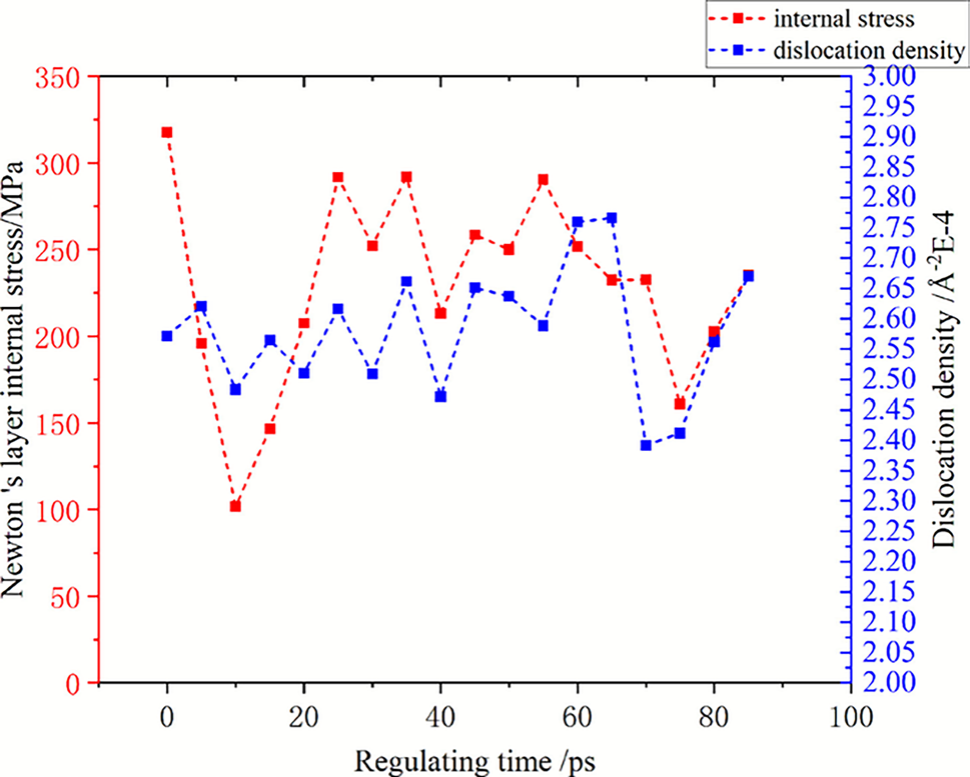
Evolution of internal stress and dislocation density during high energy acoustic beam control.

The hydrostatic stress at the time of complete relaxation before and after regulation is regarded as the residual stress, and the total dislocation length at each time is counted. The changes of stress and dislocation density during the regulation process are shown in Fig. 11. The residual stress of the initial Newtonian layer is 317.7 MPa. After self-relaxation, a significant ultrasonic softening phenomenon occurs immediately when a high-energy acoustic beam is applied to the material, that similar to the simulation results of Zhao et al.^33^. The stress decreases to 195.8 MPa within 5 ps, and the internal stress reaches a minimum of 101.9 MPa after 10 ps. After that, the internal stress gradually increased and showed a fluctuating trend, and the overall internal stress was 202.8 MPa at the end of 80 ps regulation. After relaxation, the stress tends to be balanced as a whole, and the final average residual stress is 235.3 MPa, which is 25.9% lower than the initial residual stress. In the process of high-energy acoustic beam regulation, the stress inside the material will decrease rapidly, and then oscillate within a certain range. After unloading the high-frequency vibration, the internal stress will rise again and return to a relatively stable state. After the final regulation, the average residual stress can be effectively reduced.

Combined with the variation of internal stress, the evolution of dislocation density can be divided into two stages. In the first stage, the initial energy injection material first causes the whole atom to bear the high-frequency alternating load. The sum of the residual stress inside the material and the external load exceeds the yield strength to undergo new plastic deformation, so that the overall dislocation density increases slightly. At this time, all kinds of accumulated dislocations absorb energy to activate movement, slip and small elastic deformation occur to balance the internal stress. In the second stage, with the increase of the energy of the whole system, the ultrasonic softening effect plays a leading role. The movement, proliferation and annihilation of dislocations directly affect the magnitude of internal stress. Finally, the residual stress after self-balancing of internal stress is also positively correlated with dislocation density. When the amplitude is 3 Å, the change trend of dislocation density is almost completely consistent with the change of internal stress. The stress increases with the increase of dislocation density, and the distribution of dislocation density after relaxation is also consistent with the distribution of residual stress.

**Fig. 12:**
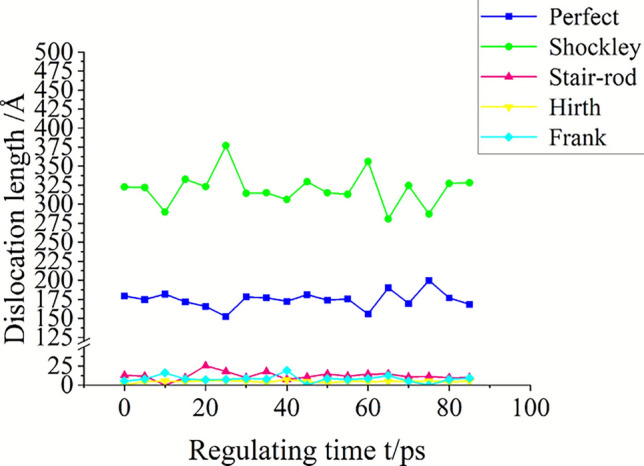
The length variation of different types of dislocations during the regulation process.

The evolution of microscopic dislocations during the ultrasonic regulation process directly corresponds to the gradual release of residual stress. In the 7050 Al alloy after milling, the predominant crystal defects are dislocations, accompanied by a limited number of distorted atoms and vacancy point defects. As shown in Fig. 12, the length changes of all dislocation types throughout the regulation process were statistically analyzed. Within the material, Shockley partial dislocations are the most abundant, followed by perfect full dislocations. Hirth dislocations, Frank dislocations, and immobile stair-rod dislocations are comparatively scarce. The Shockley partial dislocations, characterized by a small Burgers vector, are the most susceptible to slip under the energy input from ultrasonic regulation. Consequently, their length evolution trend is nearly identical to the change in total dislocation density, making them the primary factor directly governing the final state of residual stress after regulation. In contrast, perfect full dislocations can dissociate under high-amplitude loading to form new Shockley partial dislocations, which explains why their length trend is essentially opposite to that of the Shockley dislocations.

### The release mechanism of milling residual stress

**Fig. 13:**
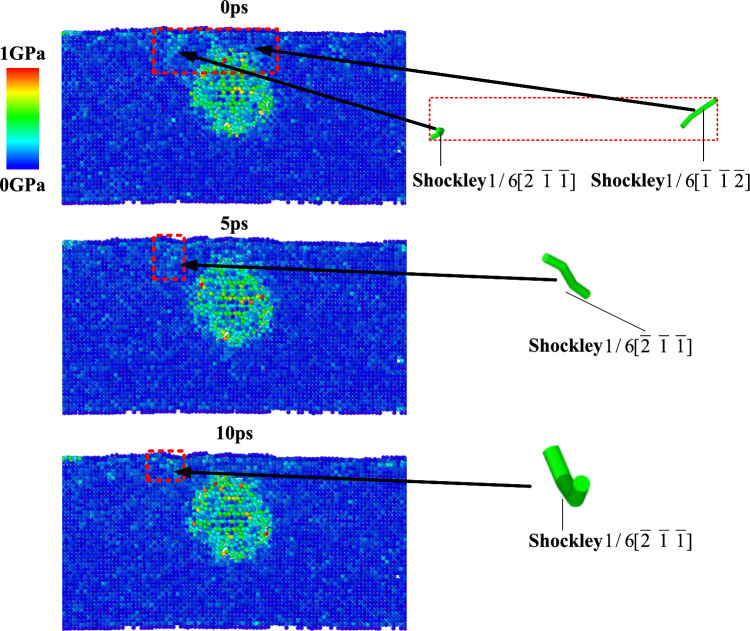
Dislocation morphology and Mises stress distribution in the early stage of regulation.

The central atomic layer with a width of 10 Å was taken from the XOZ plane of the model, and the dislocation distribution and Von Mises stress cloud diagram at the first stage of regulation (0–10 ps) were drawn as shown in Fig. 13. The lattice where dislocations nucleate and slide under shear stress is a slip zone, which causes the internal lattice atoms to deviate from the equilibrium position to produce lattice distortion. The constraint force inside the lattice has a constraint on the atoms deviating from the equilibrium position so that they always move to the equilibrium position, resulting in an internal stress field. In 7050 aluminum alloy, the volume difference between η-phase and FCC matrix atoms leads to its compression, resulting in tensile residual stress, and a large yield plastic deformation zone is formed at the center of the model. At the same time, after milling, a plastic deformation layer was formed above the η-phase, and Shockley partial dislocations in two different directions were retained. In the early stage of ultrasonic regulation, the total dislocation density fluctuates little, and the internal stress drives the dislocation morphology to change under the action of superposition effect, and gradually starts to move. All atoms also have periodic vibration transmission ability, so that the internal stress changes. It can be seen that when the control time is 5 ps, the Shockley partial dislocation of 1/6 $[\overline{1} \overline{1} \overline{2} ]$ has moved out of the observation area, while the Shockley partial dislocation of 1/6 $[\overline{2} \overline{1} \overline{1} ]$ has also undergone micro-deformation while sliding slightly, which changes the stress distribution of the surrounding atoms. That is to say, under the action of ultrasound, the slip system of dislocation will be activated first (Shockley dislocation), which will promote the dislocation to start moving, and make the dislocation itself produce small elastic deformation to rebalance the local stress.

The main conditions for the dislocation to initiate motion on the slip plane and the slip crystal depend on whether the shear stress component of the external load acting on the slip system is greater than the magnitude of the lattice resistance (Peierls stress) that the dislocation needs to overcome to cross the perfect lattice. The critical shear stress $\tau_{P - N}$ required for dislocation motion is called Peierls-Nabarro force, and its calculation formula is:

14 $$\tau_{P - N} = \frac{2\pi }{{b^{2} }}E = \frac{2G}{{(1 - \nu )}}\exp \left( {\frac{{ - 2\pi \omega_{dis} }}{b}} \right)$$

Among them, the $\omega_{dis}$ is dislocation width. That is, in the case of ultrasonic regulation, the vibration shear stress provided by the acoustic energy to the internal lattice is greater than the Peierls-Nabarro force required for dislocation motion, which can promote its motion slip.

**Fig. 14:**
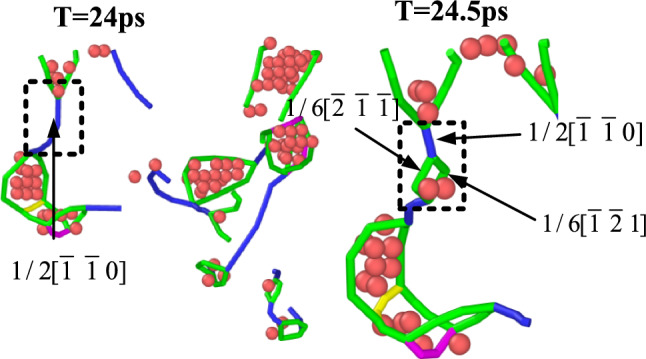
Perfect full dislocation is decomposed into Shockley partial dislocation.

After entering the second stage, the various proliferation and annihilation reactions of dislocations dominate, and have the greatest influence on the release of stress. It can be seen from Eq. 13 that the longer the total dislocation length L contained in the dislocation pileup group, the more the number of dislocations in the whole pileup group N, which also shows that the evolution relationship between the length of Shockley partial dislocation and the internal stress tends to be consistent during the regulation process. When the length decreases, the number of corresponding dislocations decreases, the overall dislocation density decreases, and the internal stress decreases. The process of ultrasonic control is essentially a process of mechanical wave propagation in solids. When a sound wave with higher energy passes through a crystal atom, it is bound to cause greater disturbance. After the milling process, a large number of dislocations are activated by the external force under the action of the ultrasonic energy field and begin to react, as shown in Fig. 14. A more typical reaction is that the Perfect dislocation can be decomposed into two Shockley partial dislocations under sufficient energy activation. The newly generated Shockley dislocations have the same name and the orientation of the Burgers vector is different, so they repel each other and gradually separate under the action of repulsive force. At the same time, the Shockley partial dislocation is formed by the stacking of atoms. The dislocation line and the Burgers vector are on the (111) slip plane, and the slip is most likely to occur. In the process of regulation, two different sign Shockley dislocations will attract and annihilate each other. Similarly, the Stair-rod dislocation will further undergo the following decomposition reaction under the action of high-energy sound field.

**Fig. 15:**
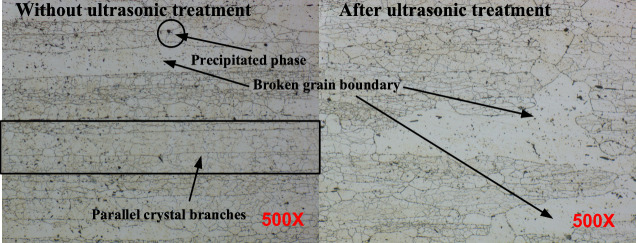
The comparison of metallography before and after ultrasonic regulation of 7050 Al alloy.

Other research teams have found that the change of stress is directly related to the movement and reaction of dislocations by applying ultrasonic vibration similarly^37,38^. We have also previously found that the dislocation density in the titanium alloy test block after ultrasonic regulation is significantly reduced by XRD characterization^39^. In order to further verify the effect of ultrasonic regulation on the microstructure of 7050 aluminum alloy, we used the GX53 optical microscope produced by Olympus company to observe the microstructure of the unregulated test and the sample treated with 100 W for 15 min after milling. According to GB/T 3246.1-2012 operation, the use of hydrofluoric acid, hydrochloric acid, nitric acid, water mixing. The etching solution is used to pretreat the 30 s chemical etching of metallographic test, and the results are shown in Fig. 15. After ultrasonic treatment, the distribution of parallel dendrites in the sample is destroyed, and a larger area of broken grain boundaries appears. This side reflects that the micro plastic deformation also occurs inside the material as the stress is released. Consistent with the analysis in Sect. 3.3, the relatively aggregated precipitates become more dispersed than before ultrasonic regulation, so their hindrance to dislocations is also reduced, which is more conducive to dislocation movement and reaction.

In summary, plastic deformation induced by milling is directly manifested on the microscopic scale by the number, size, and distribution of dislocations, whose reactions and collective motion constitute the very mechanism of plastic deformation. The fundamental principle by which ultrasonic treatment reduces and homogenizes residual stress lies in the energy absorption and consequent activation of dislocations within the applied high-energy field. This activation enables dislocations to overcome their intrinsic potential energy barrier for motion, leading to slip and localized elastic deformation that rebalances the internal stress. With the continuous input of ultrasonic energy, extensive annihilation reactions occur predominantly among the Shockley partial dislocations, causing a net decrease in total dislocation density. Concurrently, the crystal lattice recovers from a distorted, high-energy state to a more perfect, lower-energy configuration. Upon cessation of the ultrasonic regulation, the internal stress of the material is relaxed and released through this dislocation-mediated plastic deformation, resulting in a stable, low-energy state.

## Ultrasonic treatment stress parameter selection test

**Fig. 16:**
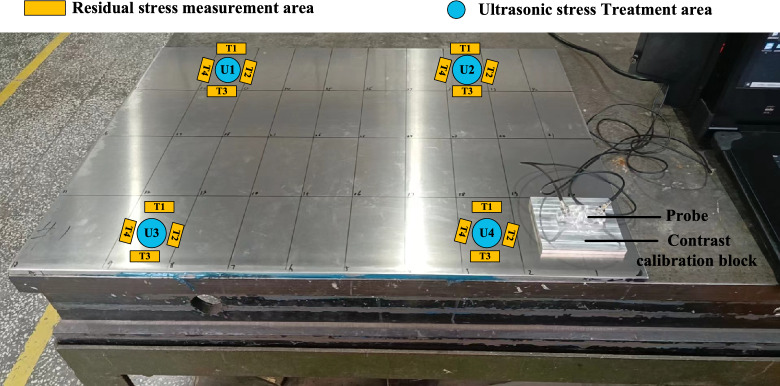
Schematic diagram of residual stress treatment and measurement.

At present, various studies have shown that when the ultrasonic transducer is directly in contact with the surface of the metal component, the control treatment can effectively reduce and homogenize the local residual stress, thereby improving the overall stress distribution of the component^40,41^. We also have previously verified the stress relief of small aluminum alloy plate parts by ultrasonic control method. Through the long-term treatment of multiple transducers, the machining residual stress of the parts can be effectively eliminated and the final deformation can be suppressed^42,43^. For large-size weak-stiffness aluminum alloy plate components, reasonable transducer control position and excitation time are the key to efficiently reduce residual stress and an effective way to improve product quality. So we designed a single-channel transducer ultrasonic treatment test to verify the stress relief effect of the method, which can help to reasonably select the aluminum alloy stress relief control process parameters. The actual machining deformation site often requires in-situ and non-destructive methods to quickly measure the residual stress distribution in the parts. Therefore, the critical refraction longitudinal wave method was used to measure the stress changes before and after ultrasonic regulation. We selected a common aluminum alloy pre-stretched sheet with a thickness of 20 mm for ultrasonic treatment test to determine the stress release law of a single channel transducer under different control time and control power. The position of the transducer and the stress measurement area are shown in Fig. 16. The detection instrument is an ultrasonic stress detector developed by Beijing Institute of Technology. The 5 MHz center frequency probe used in the field can detect the average stress value of the aluminum alloy blank in the depth range of 1 mm^43^. The preparation of the contrast calibration block is based on GB/T 38811, and the stress measurement operation is based on GB/T32073. The average value of three consecutive measurements at the same position is the stress value at this position.

**Fig. 17:**
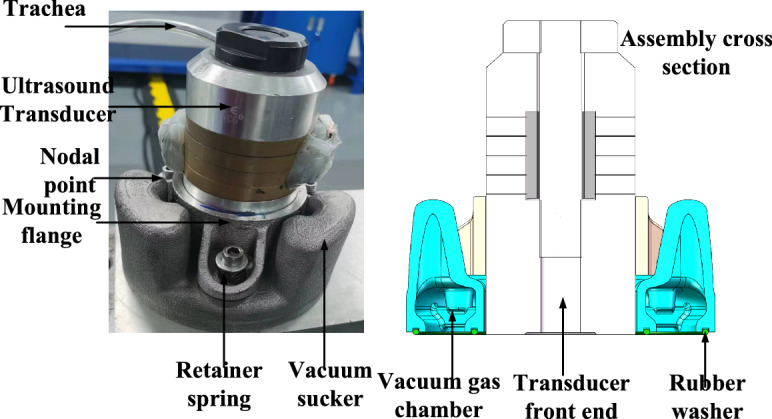
Design description of vacuum sucker device.

In order to quickly and easily control the stress at any position on the thin-walled aluminum alloy plate, a vacuum sucker device fixed ultrasonic equipment was designed, as shown in Fig. 17. During the assembly, the front end of the transducer is first mounted downward into the fixed flange, fixed at the node position, and then the fixed flange is installed on the vacuum sucker with screws and springs. The front end of the transducer is slightly higher than the rubber gasket. When it is pressed and placed on the plate, the vacuum pump is opened to vacuum the vacuum chamber, so that the whole device is adsorbed on the surface of the plate. At this time, the entire fixed flange moves upward to drive the pressing spring in a compressed state, thereby giving the transducer a constant downward pressing force.

**Fig. 18:**
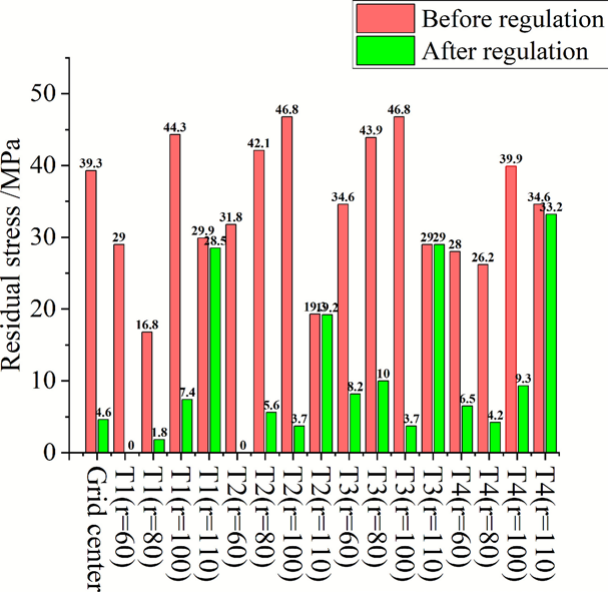
Variation of residual stress in U1 control region.

The transducer with a diameter of 55 mm and a working frequency of 20 kHz is placed at the center of the U1 grid. The residual stress changes of the transducer center position (grid center position) before and after the control and the diameter distribution of the concentric circle of 60 mm/80 mm/100 mm/110 mm with the grid center position as the origin are compared to determine the transducer control range of a single channel. The specific regulation parameters are shown in Table 4. As shown in Fig. 18, the residual stress at the position less than or equal to 100 mm from the center diameter of the transducer is greatly reduced after high-energy acoustic beam regulation, while the residual stress at the four positions with a diameter of 110 mm is almost unchanged before and after regulation. Therefore, it is considered that the effective adjustment range of a single 20 kHz transducer is a circular area with a diameter of 100 mm at the front end of the transducer.

**Table 4:** Parameter setting of high-energy acoustic beam control.

Regulatory region	Regulating time (min)	Regulate power P/W
U1	10	100
U2	5	100
U3	10	50
U4	20	100

**Fig. 19:**
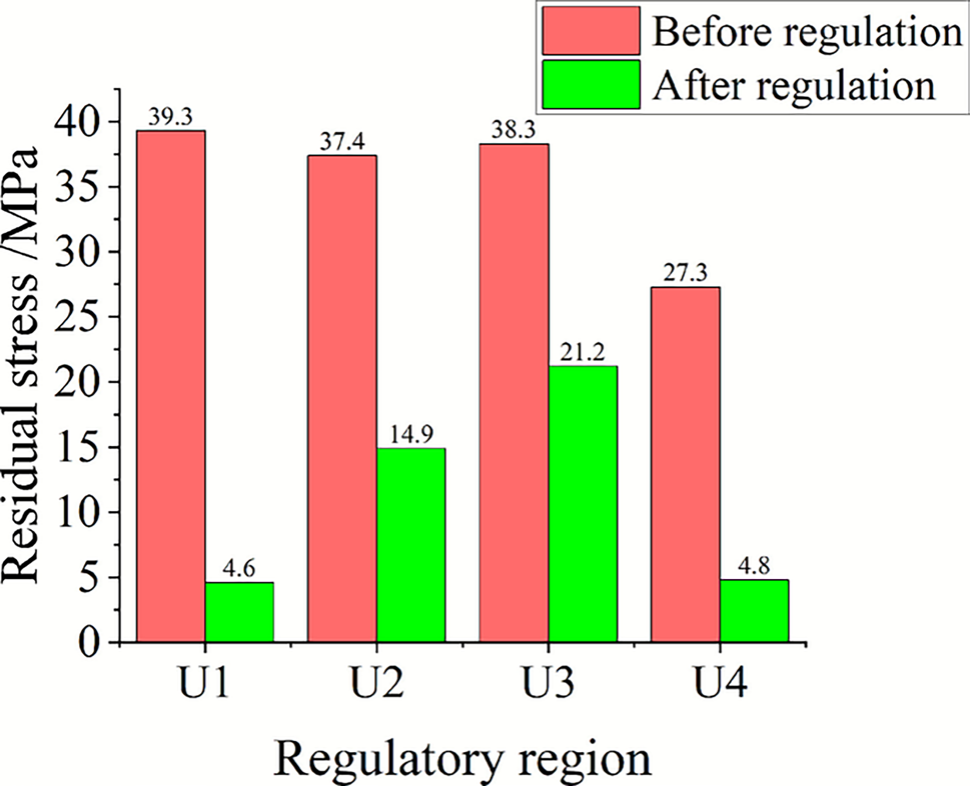
Trend diagram of residual stress reduction under different control processes.

The research shows that the acoustic beam energy applied to the material when the transducer is in direct contact with the material is related to the control time and the control power, that is, the reduction effect of the residual stress after the control is directly affected by the control time and the output power of the transducer. The change of residual stress at the center of the transducer after different control parameters in the U1 to U4 regions on the blank is compared, as shown in Fig. 19. Under the same control time (U1/U3 region), the higher the control power, the more obvious the decrease of residual stress ; when the control power is constant (U1/U2/U4 region), the residual stress decreases rapidly with the increase of the control time, and the residual stress remains at a low level after the control time exceeds 10 min. When the aluminum alloy component is actually regulated, the transducer can be set in the range of 100 mm per interval according to its initial residual stress distribution, and the higher output power can be set without damaging the surface of the component. When the residual stress of the regulated area is maintained within the range of 10 $\sigma_{{\mathrm{s}}}$%, it is considered to be in a low stress state at this time and can no longer be regulated.

## Summary

Ultrasonic stress control technology, as a method for reducing machining residual stress, has shown increasing potential in controlling the distortion of large-scale components. This study aims to elucidate the underlying mechanism of residual stress release under ultrasonic action. To this end, a molecular dynamics model incorporating η-phase precipitates was developed for the widely used 7050 aluminum alloy, enabling the continuous simulation of both residual stress introduction during milling and its subsequent release under ultrasonic treatment. The principal conclusions are as follows:

1. The primary material removal mechanism during milling with small sharpness tool is atomic shearing. This process generates significant high plastic strain zones beneath the tool and distinct shear bands ahead of it. Dislocation emission and reactions occur continuously throughout milling. The presence of η-phase precipitates impedes dislocation motion, leading to pronounced dislocation pile-ups around these obstacles. Based on dislocation pile-up theory, the concentrated residual stress after milling can be interpreted as the macroscopic manifestation of the substantial plastic strain resulting from these trapped dislocations.
2. The stress release observed during ultrasonic treatment is intrinsically governed by dislocation motion and reactions. In the initial stage, dislocations absorb energy from the ultrasonic field, becoming activated and overcoming their potential energy barrier for motion. This activation induces slip and localized elastic deformation, initiating internal stress rebalancing. With sustained energy input, extensive annihilation reactions, primarily among Shockley partial dislocations, lead to a net decrease in total dislocation density and a consequent release of internal stress. The residual stress concentration zones formed by dislocation aggregation can be equivalently regarded as intrinsic high-energy fields. Post-regulation, the system’s potential energy decreases markedly, and the lattice recovers from a distorted, high-energy state to a more perfect, lower-energy configuration. Preliminary single-channel regulation tests suggest a positive correlation between applied power and stress reduction efficacy. Furthermore, the stress reaches a stable, lower level after a certain treatment duration and remains largely unchanged thereafter.
3. For practical ultrasonic stress relief, the treatment time for a single transducer is not indefinitely proportional to effectiveness. Based on the mechanistic understanding derived from our simulations and supported by preliminary experimental trials on aluminum alloy plates, effective stress reduction to a stable lower level can be achieved by employing a moderate ultrasonic power over a finite processing duration, with transducers arranged at an appropriate interval.

## Supplementary Information

Supplementary Information.

## Data Availability

Data cannot be shared openly but are available on request from authors. Data sets generated during the current study are available from the corresponding author on reasonable request. The LAMMPS data and ultrasonic test data are available from computing server and equipment provided by author 's unit but restrictions apply to the policy requirements of the author 's unit, which were used under permission for the current study, and so are not publicly available.
