# Supplementary material for: Molecular dynamics study on the release of residual stress in milling of 7050 aluminum alloy by ultrasonic treatment
Source: Sci Rep. 2026 Feb 27;16:11291. doi: 10.1038/s41598-026-40889-6 (PMC13049100; doi:10.1038/s41598-026-40889-6)
Supplement: Supplementary file 1 — Supplementary Information. [file 41598_2026_40889_MOESM1_ESM.pdf]

Supplementary materials

1.Initial model information

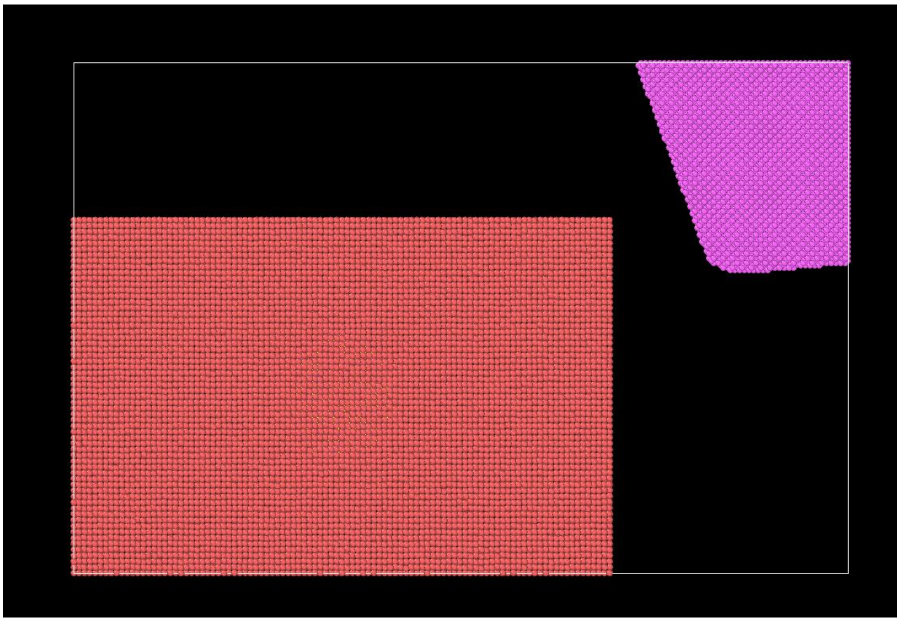

The schematic diagram of the initial model

Part of the core milling code instructions in LAMMPS

```
units      metal
dimension      3
boundary      sf pp fs
atom_style       atomic
atom_modify         map hash
neighbor               2.0 bin
neigh_modify       delay 5
newton                  on
read_data        scratch.data
region               boundary_layer      block INF 205 INF INF    INF 10 units box
region               thermostat_layer block INF 205 INF INF    10 25 units box
region               newtonian_layer    block INF 205 INF INF 25 INF units box
group               boundary_layer      region boundary_layer
group               thermostat_layer region thermostat_layer
group               newtonian_layer    region newtonian_layer
group               indenter                type 4
group               worker type 1 2 3

# ----------------------- FORCE FIELDS -----------------------------
pair_stylehybrid/overlay meam/c lj/cut 7.0
pair_coeff       * * meam/c MgAlZn.library.meam Mg Al Zn MgAlZn.parameter.meam Al Mg Zn
NULL
pair_coeff 1 4 lj/cut 0.048    3.305
```

pair_coeff 2 4 lj/cut 0.0046 3.21
pair_coeff 3 4 lj/cut 0.0048 3.31
pair_coeff 4 4 none
#Defining compute
#Defining variables
#fix XXX instruction
#write_restart instruction
….

2.MgAlZn.library.mean(Potential function file)

```
#
# Generated by MPC on 20170921T174037
#
#
# elt        lat     z       ielement     atwt
# alpha      b0      b1      b2           b3      alat    esub    asub
# t0         t1              t2           t3              rozero  ibar
#

'Mg' 'hcp' 12 12 24.305000
5.608000 2.000000 1.300000 1.300000 1.000000 3.190000 1.510000 0.520000
1.000000 5.550000 3.000000 -7.400000 1.000000 3

'Al' 'fcc' 12 13 26.981500
4.690000 3.200000 2.600000 6.000000 2.600000 4.050000 3.360000 1.160000
1.000000 3.050000 0.510000 7.750000 0.500000 3

'Zn' 'hcp' 12 30 65.380000
6.950000 1.80000 2.500000 5.300000 6.500000 2.78500 1.325000 0.700000
1.000000 25.000000 -17.300000 51.500000 1.000000 3
```

3. MgAlZn.parameter.mean(Potential function file)

```
Cmax(1,1,1)=2.900000
Cmin(1,1,1)=0.490000
Cmax(2,2,2)=2.800000
Cmin(2,2,2)=0.490000
repuls(2,2)=0.100000
Cmax(3,3,3)=2.500000
Cmin(3,3,3)=1.200000
repuls(3,3)=0.100000
attrac(3,3)=0.100000
Cmin(1,1,2)=0.490000
```

Cmin(2,2,1)=0.490000
Cmin(1,2,1)=0.360000
Cmin(1,2,2)=0.360000
Cmax(3,3,1)=2.000000
Cmin(3,3,1)=0.300000
Cmin(1,3,3)=1.000000
Cmin(1,1,3)=1.0
nn2(1,1)=1.000000
nn2(2,2)=1.000000
nn2(3,3)=1.000000
nn2(1,2)=1.000000
nn2(1,3)=1.000000
nn2(2,3)=1.000000
rho0(2)=1.175000
lattce(1,2)='l12'
Ec(1,2)=2.067960
re(1,2)=3.096460
alpha(1,2)=5.016740
rho0(3)=0.840000
lattce(1,3)='l12'
Ec(1,3)=1.470000
re(1,3)=3.040000
alpha(1,3)=5.710000
lattce(2,3)='l12'
Ec(2,3)=2.84
re(2,3)=2.856
alpha(2,3)=4.57
rc=6.000000
ialloy=0.000000
augt1=0.000000
delr=0.100000
emb_lin_neg=1.000000
bkgd_dyn=1.000000
repuls(2,3)=0.075000
Cmin(2,2,3)=0.360000
Cmin(3,3,2)=2.000000
attrac(2,3)=0.750000
